# A Smart Colorimetric Platform for Detection of Methanol, Ethanol and Formic Acid

**DOI:** 10.3390/s22020618

**Published:** 2022-01-13

**Authors:** Mizaj Shabil Sha, Muni Raj Maurya, Mithra Geetha, Bijandra Kumar, Aboubakr M. Abdullah, Kishor Kumar Sadasivuni

**Affiliations:** 1 Center for Advanced Materials, Qatar University, Doha P.O. Box 2713, Qatar; misajmisfa@gmail.com (M.S.S.); muni88_raj@yahoo.co.in (M.R.M.); geethmithramg@gmail.com (M.G.); bakr@qu.edu.qa (A.M.A.); 2 Department of Technology, Elizabeth City State University, Elizabeth City, NC 27909, USA; bkumar@ecsu.edu

**Keywords:** carbon dioxide, electrochemical conversion, methanol, ethanol, formic acid

## Abstract

Carbon dioxide (CO_2_) is a greenhouse gas in the atmosphere and scientists are working on converting it to useful products, thereby reducing its quantity in the atmosphere. For converting CO_2_, different approaches are used, and among them, electrochemistry is found to be the most common and more efficient technique. Current methods for detecting the products of electrochemical CO_2_ conversion are time-consuming and complex. To combat this, a simple, cost-effective colorimetric method has been developed to detect methanol, ethanol, and formic acid, which are formed electrochemically from CO_2_. In the present work, the highly efficient sensitive dyes were successfully established to detect these three compounds under optimized conditions. These dyes demonstrated excellent selectivity and showed no cross-reaction with other products generated in the CO_2_ conversion system. In the analysis using these three compounds, this strategy shows good specificity and limit of detection (LOD, ~0.03–0.06 ppm). A cost-effective and sensitive Internet of Things (IoT) colorimetric sensor prototype was developed to implement these dyes systems for practical and real-time application. Employing the dyes as sensing elements, the prototype exhibits unique red, green, and blue (RGB) values upon exposure to test solutions with a short response time of 2 s. Detection of these compounds via this new approach has been proven effective by comparing them with nuclear magnetic resonance (NMR). This novel approach can replace heavy-duty instruments such as high-pressure liquid chromatography (HPLC), gas chromatography (G.C.), and NMR due to its extraordinary selectivity and rapidity.

## 1. Introduction

Carbon dioxide is a gas that occupies 0.03% of the air volume. The release of large amounts of CO_2_ into the atmosphere every day in many ways contributes to the greenhouse effect, which warms the atmosphere to provide the conditions needed to sustain life. It is estimated that by 2100, anthropogenic CO_2_ levels may reach 590 ppm, increasing global temperatures by 1.9 °C [1]. The Intergovernmental Panel on Climate Change (IPCC) has recommended 350 ppm CO_2_ as the maximum safe level for atmospheric greenhouse gas concentrations. The Paris Agreement aims to decrease atmospheric CO_2_ levels by 2050 in most countries [2,3]. CO_2_ conversion, therefore, becomes a viable option to address this without interfering with the development of the urbanization process [4]. The products of CO_2_ conversion can also be used for industrial chemicals production or energy production. As a result, this can complete the carbon cycle (C-cycle) and generate energy while reducing greenhouse gases in the environment [5]. María J et al. designed a novel process that produces green gasoline on an industrial scale [6]. Qingchun Yang et al. proposed four co-feed processes of coal and coke oven gas to ethylene glycol with different methane reforming technologies: steam reforming, dry reforming, combining steam and dry reforming, and tri-forming of methane technologies [7]. i-Suhailah Rosli et al. proposed modeling the attached growth of *Chlorella vulgaris* onto polyurethane foam support material in a fluidized bed bioreactor while simultaneously bioremediating real nutrient-rich wastewater and bio fixing CO_2_ for biodiesel production [8].

Sustainable societies require that chemical commodities be obtained in an environmentally friendly and energy-efficient way. CO_2_ conversion into value-added chemicals has a clear long-term goal [9]. However, this can only be achieved using highly active and selective catalytic processes under favorable circumstances [10]. CO_2_ conversion products can supplement chemical feedstocks used in industry and cut the cost of transportation. CO_2_ has its advantages, including affordability and nontoxicity, renewable energy sources, increased productivity, and cost-effectiveness [11]. Despite its scientific challenges, carbon dioxide conversion has significant benefits. Chemically and thermodynamically, CO_2_ is a very stable molecule. Consequently, CO_2_ conversion reactions are endothermic and require efficient catalysts to achieve high yields [12]. Various strategies have been used to convert CO_2_, including thermal catalysis, photocatalysis, electrocatalysis, photoelectrochemical reactions, thermocatalysis, radiolysis, and geological storage [13].

Electrochemical reduction of carbon dioxide is typically achieved by coating an electrode with catalytic ink. Electrons are transferred by applying a sufficient voltage. Faradaic efficiency, overpotential, and current density are the most relevant metrics for CO_2_ conversion. Carbon dioxide chemical bonds can be broken by electron and proton pairs [14]. Various compounds, including hydrocarbons, can be produced via CO_2_ conversion, depending on the catalyst used. Copper electrodes can electrochemically reduce CO_2_ to form C_1_ products (formic acid and methanol) and C_2_ products (ethanol) [15]. The compounds can be detected using classic chemical, enzymatic, chemo sensing, and biosensing methods [16,17,18,19]. Formic acid (formate) is the main reason for toxicity and death through methanol poisoning. The simultaneous determination of methanol, ethanol, and formate in the body can help discover the cause of death and is useful in diagnosing acute methanol poisoning [20].

Current methods are not selective or sensitive enough, and some are extremely costly. These methods require external detection of the species that result from CO_2_ reduction. In terms of dynamics, ex situ detection has been found challenging to analyze. Therefore, it is imperative to develop sensitive and specific methods for detecting these compounds [21]. Colorimetry involves replacing subjective responses to colors with an objective numerical system [22,23]. Colorimetry quantitates the nature of the illumination and the object’s optical properties, leading to RGB color representations [24]. Compared to other methods, this method has obvious advantages, including affordability, simplicity, and fast response [25]. 

This study proposes a colorimetric detection integrated with a simple smartphone-assisted technique based on dyes systems to provide dynamic identification of CO_2_ conversion products, such as formic acid, methanol, and ethanol, and unique mechanistic insight. The simplest oxygenates produced by CO_2_ conversion are formic acid and methanol. Formic acid, methanol, and ethanol selectivity largely depend on the reduction methods and catalysts. The research aims to determine the presence of these compounds quickly and easily by colorimetry integrated with the IoT platform.

## 2. Experiments and Methods

### 2.1. Materials and Instruments

Formic acid (99.5%), methanol (99.5%), and ethanol (99.5%) were purchased from BDH Ltd. Pool England. Acquiring potassium permanganate (99%), eosin blue (90%), phenyl red, methyl orange, methyl red, and alizaringelb G.G. was done via E. Merck, Darmstadt, Germany. Acetone (99%), DME (25%), formaldehyde (99%), salicylic acid, urea, iodine, dimethyl sulfoxide (DMSO), deuterated water (D_2_O), and sodium hydroxide were obtained from Research-Lab Fine Chem Industries, Mumbai, India for selectivity analysis and iodoform test. The experiments were conducted with deionized (DI) water from the Millipore Milli-Q water system. All reagents used in the study were of analytical grade. A Biochrom UV spectrophotometer (Biochrom Ltd., Cambridge, United Kingdom) with a 300–750 nm scanning range was used for the characterization. The electrochemical experiment was conducted with a Gamry electrochemical analyzer (reference 3000, Gamry instruments, Warminster, PA, USA). Molecular identity analysis was performed using the Spinsolve benchtop NMR spectrometer by magritek, Malvern, PA, USA.

For fabricating sensors, Arudino UNO, Adafruit LCD shield (both made in the Adafruit Industries, New York, NY, USA), LEDs (peak around 606 nm), resistors, TSL230R light-to-frequency sensor, protoboard, conductors (Cat 5 cable), and Black ABS or PLA filament were used. The case was printed by a QIDI 3D printer (Zhejiang QIDI Technology Co., Ltd., Ruian, China) to protect sensing elements.

### 2.2. Methods

#### 2.2.1. Dye Preparation and Analysis

The solutions of KMnO_4_, eosin blue, phenyl red, methyl orange, methyl red, and alizaringelb G.G. were prepared at a concentration of 0.003 M and used throughout the experiment. The pH effect of the different test analytes with different dye solutions was analyzed in acidic (2, 4, 6), neutral, and basic (9, 12) solutions. Each dye solution (10 mL) was treated with 1 mL of 0.5 ppm test solution (ethanol/formic acid/methanol) separately in the adjusted pH. Changes in color and corresponding response time were observed. These test solutions’ concentration and temperature effects in selected dyes were investigated with visible color change at a certain pH (which varied for different dyes). The detection limit of the dyes was monitored with 0.05–15 ppm concentrations of test solutions, and temperature effect was studied at 25 °C, 50 °C, 75 °C, and 100 °C. The iodoform test was performed to identify the detected compound, as some dyes can detect both methanol and ethanol. During the analysis, 0.5 M iodine solution (25 mL) and 1 M sodium hydroxide (10 mL) were added to 10 mL of dye solution containing 0.5 ppm methanol or ethanol. Selectivity analysis was performed using 0.5 ppm of major compounds from each class of organic molecules (acetone for ketones, dimethyl ether (DME) for ethers, formaldehyde for aldehydes, salicylic acid for acids, and urea for amides) at room temperature.

#### 2.2.2. Fabrication of Sensor Prototype

**Figure 1:**
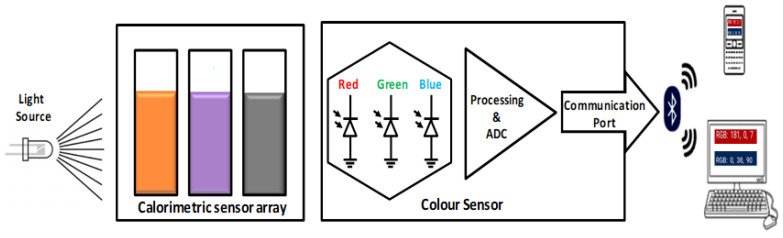
Fabricated sensor prototype for colorimetry.

A sensitive IoT-based colorimetric sensor prototype was developed to apply this novel technique, and the schematic representation of the prototype is shown in Figure 1. Three cuvettes were placed in series for the setup, and three different dye solutions were employed as sensing elements in these cuvettes. A vital light source with four lights (red, blue, white, and green) was placed at one end, and a color detector was placed at the opposite end. An Arduino nano-controlled illumination of the light source was used via an external switch. The subsequent triggering of the external switch sequentially illuminates the test chamber with white, blue, red, and green light. A portable battery powers the detector, and the RGB data is acquired using a mobile application. The detector identified color changes in dye solutions placed inside the cuvettes upon exposure to test solutions. It showed unique RGB values in the device connected to the sensor prototype through Bluetooth.

#### 2.2.3. Characterization, Electrochemical Analysis, and Quantification

A UV–Vis spectrophotometer performed all the characterization. Electrochemical measurements of the CO_2_ conversion were conducted using 0.5 M of Na_2_HCO_3_ for 15 min at room temperature. The counter, reference, and working electrode were platinum wire, Ag/AgCl (3 M KCl solution), and copper electrode, respectively, with a diameter of 5 mm. Amperometric measurements were conducted with a potential range of 0.5–0.8 V vs. a reference electrode and a sweep rate of 5 mV/s. The liquid products were quantified by NMR spectroscopy using 0.5 mL electrolyte (0.1 N NaOH) with 0.1 mL D_2_O (deuterated water) and 0.5 μL dimethyl sulfoxide (DMSO) as an internal standard. 

## 3. Results and Discussion

### 3.1. Detection of Formic Acid

#### 3.1.1. Response Time and pH Effect

pH effect of the test solution (0.5 ppm) at room temperature on a series of dye solutions with various pH values (2, 4, 6, 7, 9, and 12) was examined. Data were analyzed before and after adding the test solutions to the dyes at various pH values in each dye. 

**Figure 2:**
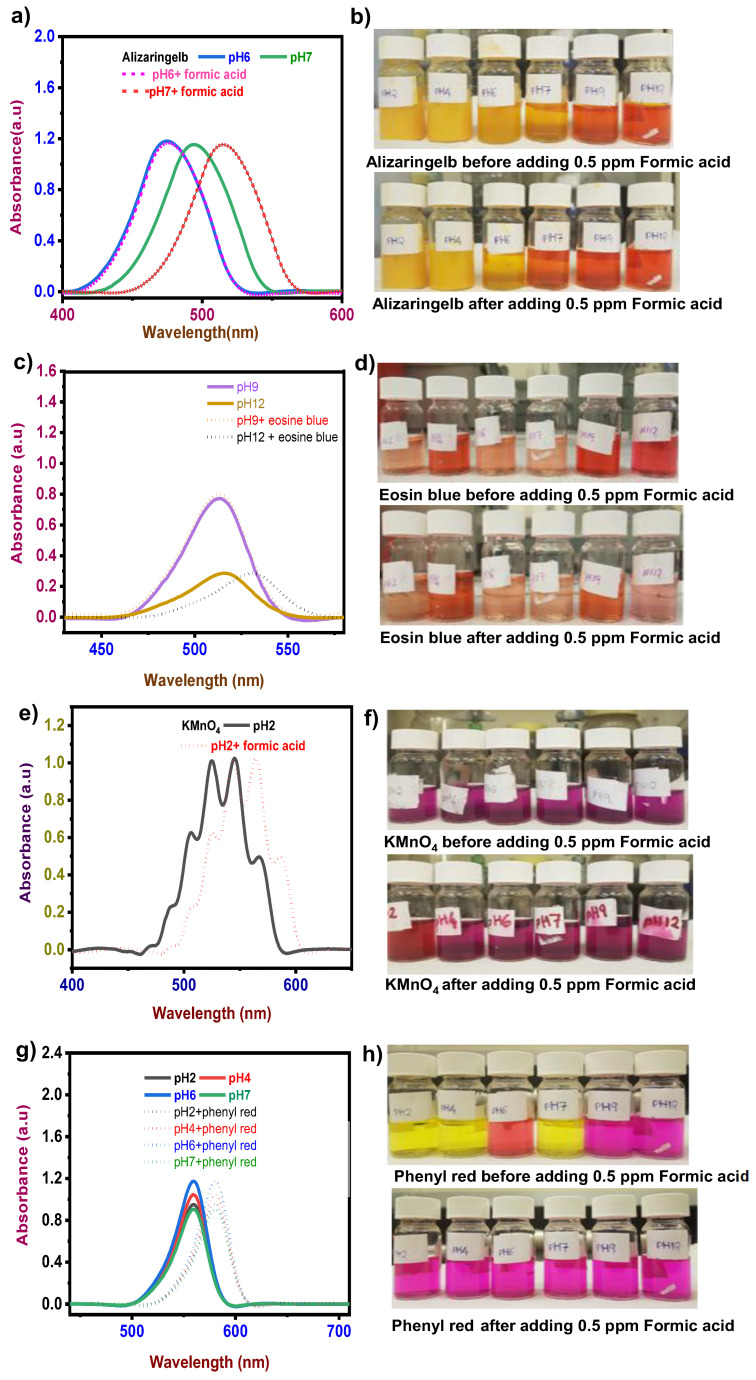
pH effect of formic acid (**a**,**c**,**e**,**g**) UVVis absorption curve of dyes in effective pH after adding 0.5 ppm formic acid. (**b**,**d**,**f**,**h**) pH adjusted dye solution at room temperature before and after adding 0.5 ppm formic acid.

In the alizaringelb dye solution, a significant visible color change was noticed in pH 6 and 7 after adding 0.5 ppm formic acid (Figure 2b). The color changed from yellow to light yellow in pH 6 and yellow to orange in pH 7. It was confirmed by the emergence of a new absorption band centered at ~474 nm from ~476 nm in the case of pH 6 and ~514nm from 495 nm for pH 7 in UV–Vis absorbance spectra (Figure 2a). A color change was observed in pH 9 and 12 eosin blue dye solution after adding 0.5 ppm formic acid (Figure 2d). Color change from red to orange in pH 9 and from red to pink in pH 12 was observed, and as a result, a slight decrease in absorbance variation at ~513 nm was observed for pH 9. For pH 12, the new absorption band with the absorbance of 0.2 a.u. centered at ~532 nm from ~516 nm was detected in the UV–Vis absorption spectrum (Figure 2c).

In the case of KMnO_4_, the bare dye solution exhibited a violet color in all pH, and after the addition of 0.5 ppm formic acid at pH 2, showed a color shift to red (Figure 2f), which was also confirmed by the new absorption band centered at ~564 nm from ~545 nm with the absorbance of 1.02 a.u. (Figure 2e). Two absorption peaks appeared in KMnO_4_. This phenomenon is common for highly conjugated systems. However, it is often solvent-dependent and might be due to electronic transitions between the different vibrational energy levels possible for each electronic state [26]. Phenyl red dye solution changed dramatically in color when formic acid was added at 0.5 ppm to pH 2, 4, 6, and 7 (Figure 2h). The color change was confirmed by the appearance of new absorption bands centered at ~581 nm from ~560 nm in all the effective pH without any variation in absorbance as it was with bare dye solution, at pH 2, 4, 7 and pH 6 with the test solution (Figure 2g).

These UV–Vis analyses showed a bathochromic effect after adding test solutions in each dye at a certain pH [27]. The change in absorbance to a longer wavelength might be caused due to presence of the auxochrome (test solution), which may change in solvent polarity. The observable color change offers a convenient approach to detecting formic acid via unaided eyes. Alizaringelb, eosin blue, phenyl red, and potassium permanganate efficiently detected formic acid with a short detection time. Compared to the other two dyes, phenyl red and alizaringelb detect formic acid extremely rapidly (Table 1). Methanol and ethanol analyses in these dyes demonstrated excellent specificity and selectivity for formic acid alone.

Eosin blue is a dibromo dinitro derivate of fluorescein. Such molecules composed of two similar atoms or groups of atoms unite with a conjugated system of double carbon linking at the one and four positions. The reactions are pH-specific, but the union does not follow Thiele’s law. This might account for the color change, which occurred only at pH 9 and 12 after adding formic acid and eosin blue, from red to orange and pink, respectively.

Phenol red changes from yellow to red over pH 6 and then turns to magenta above neutral pH. The phenol red dye may react to form complexes, causing a pH change in the reaction system with formic acid, resulting in a color change. Phenol red loses its proton if the pH level in the reaction system is above 6 (pKa = 1.2), resulting in the red, negatively charged ion. The color change from red to magenta occurs at a pH that is still higher than neutral (pKa = 7.7). In the presence of formic acid, the absorbance intensity of potassium permanganate decreased, indicating that formic acid complexes form due to oxidation in which the acid molecule might act as a reactant with potassium permanganate.

#### 3.1.2. Concentration Effect

**Figure 3:**
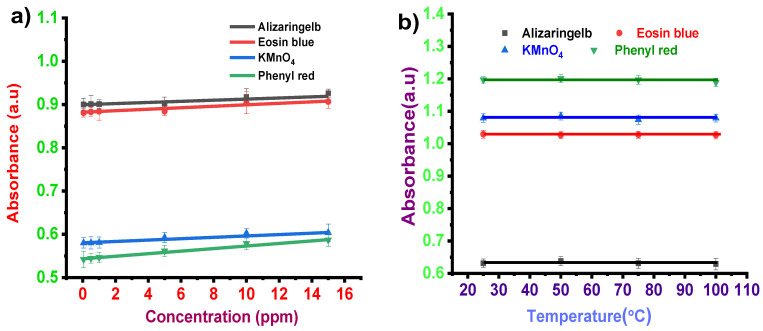
(**a**) Sensitivity analysis of formic acid in dye solutions. (**b**) Stability analysis of formic acid in dye solutions.

To analyze the sensitivity of the dyes towards the test solution, the UV spectrometric study was carried out by varying the test analyte concentration from 0.05–15 ppm in particular reactive pH of the dyes at ambient temperature. The corresponding calibration plot for estimating the dye limit of detection (LOD) towards each test analyte sensing is shown in Figure 3a. The calibration curve was plotted by considering the peak absorbance of each dye for different concentrations of specific test analytes. The absorbance peak for different dyes was 495 nm for alizaringelb, 513 nm for eosin blue, 564 nm for KmnO_4_, and 581 nm for phenyl red. Linear fitting was performed in the range of 0.05 ppm to 15 ppm to estimate the LOD using the equation

(1) $$\mathrm{LOD}=\frac{3\sigma }{m}$$

where m represents the slope of the calibration plot and σ is the standard deviation of the intercept. The linear fitting estimated an LOD of alizaringelb as ~0.059 ppm, R^2^ = 0.997. Similarly, the LOD of eosin blue for formic acid was ~0.053 ppm with R^2^ = 0.927. In the case of KmnO_4_ for different concentrations of formic acid in the range of 0.05–15 ppm, the estimated LOD was ~0.051 ppm, R^2^ = 0.947. The estimated LOD of phenyl red was ~0.060 ppm, R^2^ = 0.977. The sensitivity investigation indicates that all these dyes exhibit a high sensitivity towards formic acid with a linear detection limit as low as ~0.05 ppm in the selected concentration range (Figure 3a). This concentration study indicated that these dyes exhibit high sensitivity towards formic acid even at low concentrations. Figure 3a shows that absorbance increases, following the Lambert–Beer law, as concentration increases. Table 2 represents the response time taken by different dye solutions at reactive pH for different concentrations of formic acid varying from 0.05–15 ppm.

#### 3.1.3. Temperature Effect

One of the most important properties for a sensing application is the system’s stability towards the change in temperature of the surrounding medium. Thus, to investigate the stability of the dye’s response to the change in temperature, the dye solutions were subjected to different temperatures, and sensing towards the corresponding compounds was analyzed. To understand the effect of temperature, test solution (0.5 ppm)–dye mixture at effective pH was heated at different temperatures: 25 °C, 50 °C, 75 °C, and 100 °C. In general, for a proper sensing mechanism, the temperature effect should be negligible in the dye system. Figure 3b shows the effect of temperature on the sensing performance of selected dyes towards 0.5 ppm of test solutions. It is noticeable that the absorbance of all the dyes treated with different temperatures did not exhibit any deviation during stability analysis. Response time was almost the same for dye solutions at different temperatures.

#### 3.1.4. Selectivity

**Figure 4:**
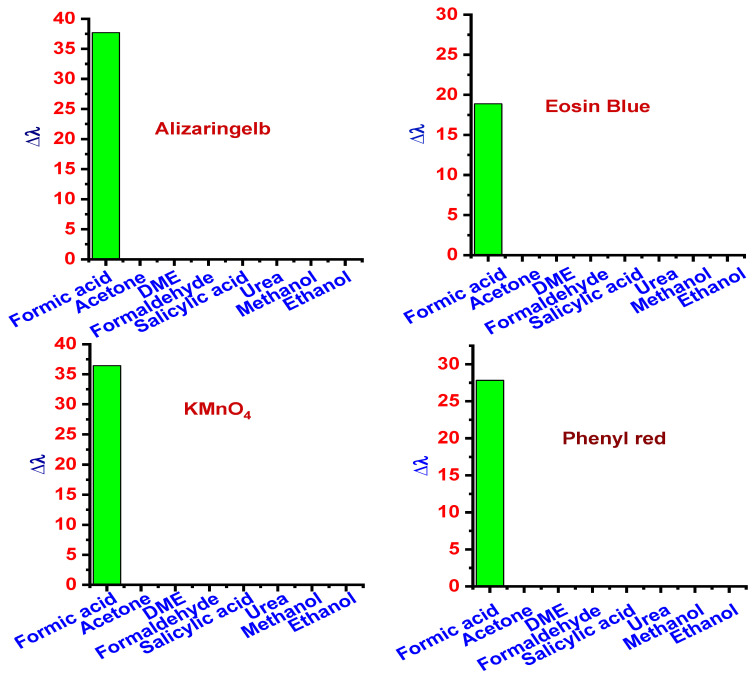
Selectivity analysis of formic acid in alizaringelb, eosin blue, KMnO_4_, and phenyl red.

One of the most important characteristics of a detection device is its ability to detect the target selectively. The selectivity of the proposed platform was tested with possible interfering molecules. All the six dyes, namely alizaringelb G.G. (pH 7), potassium permanganate (pH 2), eosin blue (pH 9), phenyl red (pH 7), methyl orange (pH 9), and methyl red (pH 9), were tested using acetone, DME, formaldehyde, salicylic acid, and urea. UV analysis showed that the four other dyes could detect formic acid except for methyl red and orange. The visible color change was not obtained with the above compounds in methyl red and methyl orange, confirming formic acid’s selectivity with these dyes. Methanol and ethanol were detected by methyl orange and methyl red solution. Subsequently, feasibility verification of these dyes’ selectivity was confirmed by measuring the relative change in the wavelength (∆λ) from UV–Vis analysis estimated by the equation below.

(2) $$\Delta \lambda =\frac{\lambda x-{\;\lambda }_{0}}{\lambda_{0}}\times 100\;$$

where λx is the wavelength of peak absorbance measured in the presence of the analyte and λ_0_ is the wavelength of peak absorbance of the blank solution. The peak absorbance wavelength (λx) for different dyes was 495 nm for alizaringelb, 513 nm for eosin blue, 564 nm for KMnO_4_, and 581 nm for phenyl red. It is clear from Figure 4 that all the dyes have additional selectivity and specificity in detecting formic acid generated by the electrochemical conversion of CO_2_. Moreover, the above results confirmed that these dye systems could develop a colorimetric strategy.

### 3.2. Detection of Methanol

#### 3.2.1. pH Effect

**Figure 5:**
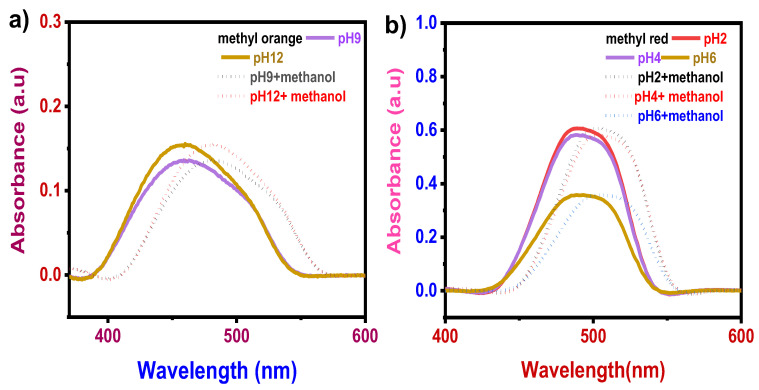
pH effect: (**a**) Methyl orange dye before and after adding methanol; (**b**) methyl red dye before and after adding methanol.

No distinct visible color change was observed in the case of methyl orange and methyl red with a test solution of 0.5 ppm methanol. Even though methanol produced no visible color change in methyl red and methyl orange, the UV–Vis analysis showed a peak shift. In the case of methanol–methyl orange mixture, new absorption bands centered at ~485 nm from ~459 nm in pH 9 with the absorbance of 0.132 a.u., as that of bare dye solution, and with pH 12, the new absorption band obtained at ~482 nm from~460 nm with the absorbance of 0.155 a.u. (Figure 5a). In the case of methanol–methyl red mixture, UV–Vis analysis peak shift was obtained at pH 2, 4 (~489 nm to ~509 nm), and 6 (~493 nm to 510 nm) with the same absorbance of 0.60 a.u., 0.581 a.u., and 0.355 a.u., respectively (Figure 5b).

#### 3.2.2. Concentration Effect

**Figure 6:**
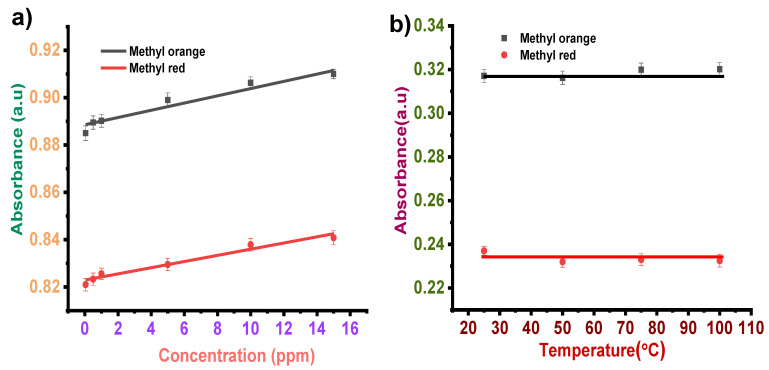
(**a**) Sensitivity analysis of methanol in dye solutions. (**b**) Stability analysis of methanol in dye solutions.

Similar to formic acid, the effect of different concentrations (0.05–15 ppm) of methanol on methyl orange and methyl red dye was examined (Figure 6a). The linear fitting by considering the peak absorbance of methyl orange at 485 nm for different concentrations of methanol estimated LOD as ~0.0375 ppm, R^2^ = 0.937. Similarly, the calibration plot of methyl red at 515 nm for different methanol concentrations estimated an LOD of ~0.0318 ppm, R^2^ = 0.955.

#### 3.2.3. Temperature Effect

Stability analysis of methanol in methyl orange and methyl red showed a negligible temperature effect. Figure 6b shows the effect of temperature on the sensing performance of methyl orange and methyl red dye solutions towards 0.5 ppm methanol.

#### 3.2.4. Selectivity Analysis

**Figure 7:**
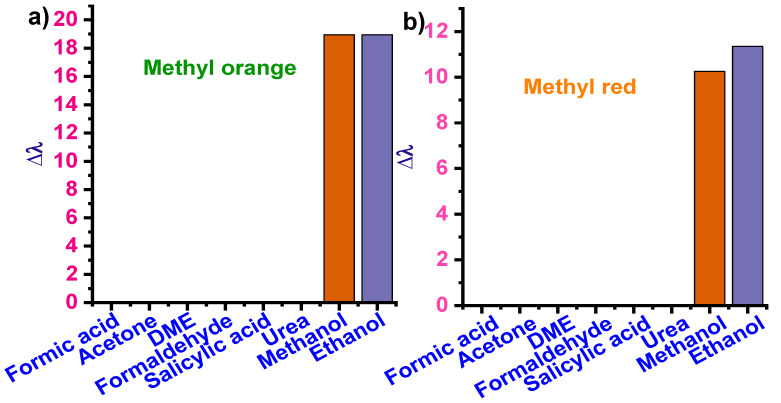
Selectivity analysis in (**a**) methyl orange and (**b**) methyl red dye solutions.

Feasibility verification of these dyes’ selectivity was confirmed by measuring the relative change in the wavelength (∆λ) from UV–Vis analysis estimated by Equation (2). Analysis was carried out in pH 9 methyl orange dye solution and pH 6 methyl red dye solution (Figure 7). It is clear from Figure 7 that these two dyes have additional selectivity and specificity in detecting methanol.

### 3.3. Detection of Ethanol

#### 3.3.1. pH Effect

In the case of methyl orange, UV–Vis analysis showed that a peak shift could be induced by ethanol in pH 7, pH 9, and 12 solution and a peak shift in pH 2, 4, 6, and 9 methyl red solutions. In the case of ethanol–methyl orange mixture, UV–Vis analysis peak shift was obtained at pH 7, 9 (~489 nm to ~509 nm), and 12 (~493 nm to 510 nm) with the same absorbance of 0.60 a.u., 0.581 a.u., and 0.355 a.u., respectively. The ethanol with methyl red results showed a bathochromic effect at 581 nm from 560 nm in pH 2, 4, 6, and 9.

**Figure 8:**
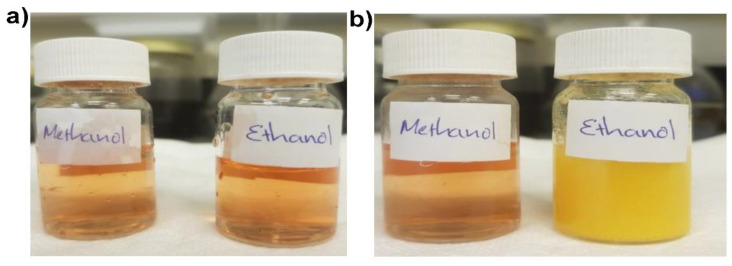
Test solutions of pH 9 methyl orange solution with methanol and ethanol (**a**) before and (**b**) after the iodoform test.

The iodoform test was used to detect carbonyl compounds, and as formic acid does not contain a carbonyl, it was not tested. The hydroxide ion removes acidic alpha hydrogen and forms an enolate ion in this reaction. An enolate anion displaces an iodide ion from the iodine molecule to produce R–CO–CI_3_. The hydroxide ion reforms the carbonyl group and eliminates the CI^3−^ anion upon bonding with the carbonyl carbon. Iodoform is precipitated in yellow color when the carboxylic acid group and the CI^3−^ ion react. The iodoform test was carried out to identify the detected compound using pH 9 solution with methyl orange and methyl red. A positive iodoform test is given by the compounds having CH_3_CO group in their structure. Thus, ethanol gives a positive iodoform test. Due to the formation of tri-iodomethane or iodoform, the solution containing ethanol became cloudy, and then a yellow precipitate formed (Figure 8). The dye solution containing methanol had no change.

#### 3.3.2. Concentration Effect

**Figure 9:**
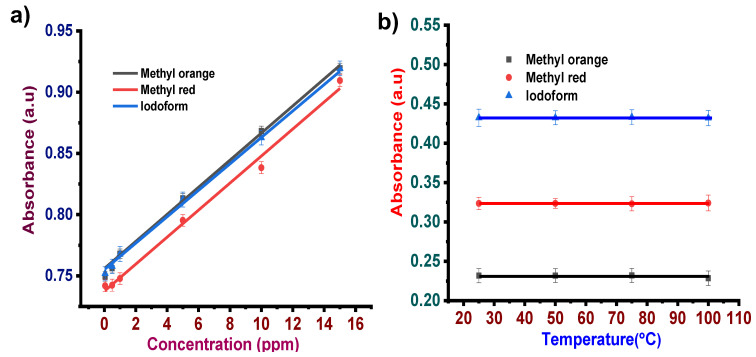
(**a**) Sensitivity analysis of formic acid in dye solutions. (**b**) Stability analysis of formic acid in dye solutions.

Sensitivity analysis of ethanol with concentration 0.05–15 ppm was carried out for methyl orange and methyl red dye solutions (Figure 9a). The linear fitting by considering the peak absorbance of methyl orange at 509 nm for different concentrations of ethanol estimated LOD as ~0.0664 ppm, R^2^ = 0.84786. Similarly, the calibration plot of methyl red at 581 nm for different methanol concentrations estimated an LOD of ~0.0614 ppm, R^2^ = 0.82671. For the iodoform test, LOD was found to be 0.0609 ppm with an R^2^ value of 0.90064. This indicates that these three systems can detect ethanol with concentrations as low as 0.0609 ppm. In the case of the iodoform test, response time was almost constant, irrespective of the change in ethanol concentration.

#### 3.3.3. Temperature Effect

Stability analysis of ethanol was carried out in methyl orange, methyl red, and iodoform at different temperatures, 25 °C, 50 °C, 75 °C, and 100 °C, and it showed negligible temperature effect. Figure 9b shows the effect of temperature on the sensing performance of the three systems towards 0.5 ppm ethanol. The temperature study reveals no significant effect of temperature on dye sensing. The above analysis revealed that this mode of dye sensing offers high stability towards the change in temperature, which is important for accurate test analyte sensing based on color development.

#### 3.3.4. Selectivity Analysis

Selectivity analysis was carried out for pH 9 methyl orange dye and pH 6 methyl red dye solutions (Figure 7). It is clear from Figure 7 that all the dyes have high selectivity in detecting ethanol.

### 3.4. Evaluation of the Proposed Colorimetric Method and Validation with NMR Technique

A portable prototype device with full functions for detecting formic acid, ethanol, and methanol was developed. Using the dyes as sensing elements, the sensor prototype showed unique RGB values upon exposure to test solutions. In the first approach, three dyes were chosen to detect each test solution at a concentration of 0.05 ppm, and the obtained RGB values were shown in Table 3.

In the second approach, the test solutions in different concentrations were analyzed using the sensor prototype in different dyes. Table 4 shows the RGB values obtained for different concentrations of formic acid in different dyes.

In the third approach, the test solutions mixture at 0.05 ppm was analyzed using different dyes, and the obtained RGB values are shown in Table 5. From the RGB chart obtained, we can predict the system of dye solutions that detected formic acid or methanol or ethanol, or all three together. A similar RGB chart can be created for different concentrations of test solutions. It was proved from these studies that we can similarly design the unique RGB chart for all dye solutions. These studies confirmed that this sensor prototype could detect the presence and the concentration of each test molecule and its concentration based on the RGB values.

Currently, related conventional detection technologies mainly include chromatography (high-performance liquid chromatography and ion chromatography), spectroscopy (spectrophotometry), biological detection, etc. [9,10,11]. However, the technologies mentioned above generally involve expensive equipment and a complicated analysis process and are unsuitable for in-field or real-time testing [28]. In an economic aspect, the choice of the proposed colorimetric method is much higher than the classical detection methods. This IoT-based prototype is cost-effective instead of these high-cost and heavy-duty instruments. The proposed method will only cost around USD 20, whereas the classic instrument costs 10 times higher.

**Figure 10:**
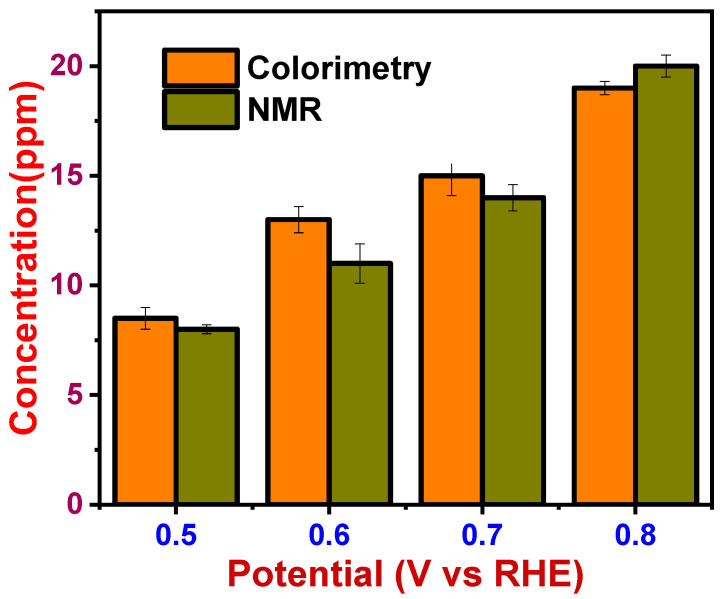
Comparison of NMR data and colorimetric data for methanol.

Furthermore, this colorimetric approach would provide results in seconds without taking hours, as would classical methods. Different analysis perspectives were carried out to determine the efficiency of the colorimetric method with the NMR technique. NMR integration results generally agree with those obtained by colorimetry, with some overestimation for related compounds probably due to peak overlap and subsequent integral errors and an apparent relative lower estimation for the target compound. Figure 10 compares the efficiency of NMR and colorimetric data for methanol.

The quantification data of methanol at the potential range of 0.5–0.8 V shows the concentration in the range of 8–19 ppm in NMR and 8.5–20 ppm in this developed sensor prototype. It is clear from these values that this new approach of colorimetric detection and quantification of formic acid, methanol, and ethanol would be a new accurate method for chemical analysis. It can replace various heavy-duty instruments used for the same purpose. The designed sensor prototype is portable and easily accessed with any Bluetooth device. The results obtained demonstrate the potential of the colorimetric method for detecting value-added products generated by recycling CO_2_.

Apart from detecting the three compounds, this IoT-based colorimetric sensor has also found other practical applications. As formic acid is the main toxic compound produced during methyl alcohol metabolism, it accumulates in the body. It is the main reason for toxic effects and death through methanol poisoning [29,30,31]. Commonly reported adverse effects of high serum concentrations of formic acid include visual damage, optical nerve injury, abdominal problems, nausea, and headache. Subsequently, high formic acid concentrations result in respiratory problems and renal failure, leading to coma and death [32]. Therefore, it seems necessary to determine the serum concentrations of methanol and formic acid to evaluate the causes of these observed adverse effects.

Similarly, ethanol is one of the breath biomarkers and is key to diagnosing and managing diabetic ketoacidosis (DKA) in patients with type 1 diabetes. It may also be of increasing importance to detect euglycemic ketoacidosis in patients with type 1 or type 2 diabetes or heart failure, treated with sodium-glucose transporter-2 inhibitors (SGLT2-i). Analysis of breath ethanol is also important for forensic purposes in diagnosing metabolic diseases and monitoring medical treatment [33].

## 4. Conclusions

In summary, we proposed a novel IoT-based colorimetric strategy for detecting methanol, ethanol, and formic acid obtained after the conversion of CO_2_. In this approach, the dye solutions act as sensing elements, and these multisensors help us provide more accurate results. From the RGB chart obtained, we predicted the system of dye solutions that detected formic acid or methanol or ethanol, or all three together, and the concentration of the three compounds. The developed method exhibited excellent response with a short response time of around 2 s and high selectivity with a low detection limit of 0.03–0.06 ppm towards detected compounds. This advanced colorimetric method is cost-effective and straightforward. These studies prove that it is possible to design a unique RGB chart for all dye solutions to their specific test analyte. While investigating the comparative efficiency of both NMR and colorimetry for the three compounds in terms of cost and accuracy helps to conclude that this dye system-based approach is more efficient than heavy-duty instruments, it can be extended to detecting these molecules and other analytical strategies, which holds great potential for detecting different molecules in different application scenarios.

## Figures and Tables

**Table 1:** Colorimetric detection of formic acid.

Sl. No	Dye Solution	Dye Structure	Detected pH and Color Change	Response Time
1	Eosin blue	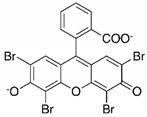	pH 9 (red to orange), pH 12 (red to pink)	10 min
2	Phenyl red	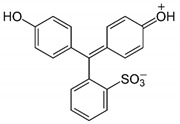	pH 2, 4, 7 (yellow to magenta) pH 6 (red to magenta)	2 s
3	Potassium permanganate	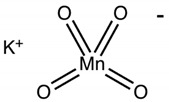	pH 2 (violet to red)	2 min
4	Alizaringelb GG	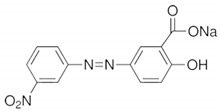	pH 6 (yellow to light yellow) pH 7 (yellow to orange)	3 s

**Table 2:** Response time of dyes for different concentrations of formic acid.

Sl. No	Dye Solution	The Concentration of Formic Acid in ppm					
		0.05	0.5	1	5	10	15
1	Eosin blue (pH 9)	-	10 min	8 min	6 min 40 s	2 min	Fraction of seconds
2	Phenyl red (pH 7)	-	2 s	Fraction of seconds	Fraction of seconds	Fraction of seconds	Fraction of seconds
3	Potassium permanganate (pH 2)	-	2 min	1 min 43 s	1 min	7 s	Fraction of seconds
4	Alizaringelb GG (pH 7)	-	3 s	Fraction of seconds	Fraction of seconds	Fraction of seconds	Fraction of seconds

**Table 3:** Analysis of F/E/M-dye mixture using sensor prototype at 0.05 ppm concentration.

Sl. No	F/E/M-Dye Mixture	RGB Values
1	A (Nil) B (Nil) C (Nil)	265, 195, 10
2	A (FX_1_) B (FX_1_) C (FX_1_)	245, 107, 80
3	A (Nil) B (Nil) D (Nil)	255, 95, 25
4	A (FX_1_) B (FX_1_) D (FX_1_)	245, 107, 60
5	G (Nil) H (Nil) I (Nil)	235, 103, 110
6	G (EX_1_) H (EX_1_) I (EX_1_)	260, 210, 115
7	G (Nil) H (Nil)	235, 118, 85
8	G (MX_1_) H (MX_1_)	275, 215, 96

A: Alizaringelb, B: Eosin blue, C: KMnO4, D: Phenyl red, G: Methyl orange, H: Methyl red, I: Iodine+ NaOH, F: Formic acid, M: methanol, E: Ethanol, X_1_ = 0.05 ppm, X_2_ = 0.1 ppm, X_3_ = 0.5 ppm, X_4_ = 1 ppm, X_5_ = 5ppm, X_6_ = 10 ppm, X_7_ = 50 ppm.

**Table 4:** Analysis of formic acid in different concentrations with the dyes using sensor prototype.

Sl. No	F/E/M-Dye Mixture at Different Concentration	RGB Values
1	A (Nil) B (Nil) C (Nil)	265, 195, 10
2	A (FX_2_) B (FX_2_) C (FX_2_)	235, 103, 40
3	A (FX_3_) B (FX_3_) C (FX_3_)	240, 116, 49
4	A (FX_4_) B (FX_4_) C (FX_4_)	246, 122, 55
5	A (FX_5_) B (FX_5_) C (FX_5_)	251, 134, 61
6	A (FX_6_) B (FX_6_) C (FX_6_)	253, 141, 66
7	A (FX_7_) B (FX_7_) C (FX_7_)	258, 147, 69

A: Alizaringelb, B: Eosin blue, C: KMnO4, D: Phenyl red, G: Methyl orange, H: Methyl red, I: Iodine+ NaOH, F: Formic acid, M: methanol, E: Ethanol, X_1_ = 0.05 ppm, X_2_ = 0.1 ppm, X_3_ = 0.5 ppm, X_4_ = 1 ppm, X_5_ = 5ppm, X_6_ = 10 ppm, X_7_ = 50 ppm.

**Table 5:** Analysis of F + E + M mixture in dyes using sensor prototype for different concentrations.

Sl. No	Solution Mixture (0.05 ppm)	Dyes	RGB Values
1	FX_1_ + EX_1_ + MX_1_	A-B-C	245, 107, 80
2	FX_1_ + EX_1_ + MX_1_	A-B-D	245, 107, 60
3	FX_1_ + EX_1_ + MX_1_	A-B-G	245, 107, 275
4	FX_1_ + EX_1_ + MX_1_	A-B-H	245, 107, 215
5	FX_1_ + EX_1_ + MX_1_	A-B-I	245, 107, 115

## Data Availability

Not applicable.
